# Supplementary material for: Live imaging of muscle histolysis in Drosophila metamorphosis
Source: BMC Dev Biol. 2016 May 4;16:12. doi: 10.1186/s12861-016-0113-1 (PMC4855724; doi:10.1186/s12861-016-0113-1)
Supplement: Additional file 1: Table S1. — List of gene perturbations tested for muscle defects in pilot screen. (PDF 120 kb) [file 12861_2016_113_MOESM1_ESM.pdf]

| Gene pertubed | Biological function | Gene perturbation Type | Construct/ TRip # | Stock Number | Prepupae scored | Lethality Stage | Eclosion [%] | Flightless rate [%] |
|---|---|---|---|---|---|---|---|---|
| **7B2** | Proteolysis, peptidase activator | RNAi | UAS-7B2-lhRNA (Long Hairpin JF02917) | B-27989 | 20 | PP 5% | 95% | 0% |
| **Activator of SUMO 1 (Aos1)** | Protein sumoylation | RNAi | UAS-Aos1-shRNA (Short Hairpin GL00493) | B-36074 | 20 | LP 25% | 75% | 0% |
| **Adenosine deaminase acting on RNA (Adar)** | RNA editing | RNAi | UAS-Adar-lhRNA (Long Hairpin JF02942) | B-28311 | 20 | | 100% | 0% |
| **Akt1** | Cell growth, organ size | Protein OE | UAS-Akt1 | B-8191 | 15 | | 100% | 0% |
| **Akt1** | Cell growth, organ size | RNAi | UAS-Akt1-lhRNA (Long Hairpin HM04007) | B-31701 | 20 | LP 35% | 65% | partial |
| **Akt1** | Cell growth, organ size | RNAi | UAS-Akt1-shRNA (Short Hairpin HMS00007) | B-33615 | 0 | BP 100% | 0% | n.d. |
| **ALG-2 interacting protein X (ALiX)** | Ubiqitin dependent proteolysis | RNAi | UAS-ALiX-shRNA (Short Hairpin HMS00298) | B-33417 | 20 | | 100.0% | n.d. |
| **AMP-activated protein kinase alpha (AMPKalpha)/SNF1A** | Protein kinase | RNAi | UAS-AMPKalpha-lhRNA (Long Hairpin JF01951) | B-35137 | 20 | | 90.0% | yes |
| **Atrophin (Atro)/Grunge** | Atrophin homolog | RNAi | UAS-Grunge-shRNA (Short Hairpin HMS00756) | B-32961 | 20 | | 95% | yes |
| **Autophagy-related-1 (Atg1)** | Autophagy | RNAi | UAS-Atg1-shRNA (GL00047) | B-35177 | 20 | | 100.0% | n.d. |
| **Autophagy-related-10 (Atg10)** | Autophagy | RNAi | UAS-Atg10-shRNA (Short Hairpin HMS02026) | B-40859 | 20 | | 100% | n.d. |
| **Autophagy-related-101 (Atg101)** | Autophagy | RNAi | UAS-Atg101-shRNA (Short Hairpin HMS01349) | B-34360 | 20 | | | n.d. |
| **Autophagy-related-12 (Atg12)** | Autophagy | RNAi | UAS-Atg12-RNAi (Short Hairpin HMS01153) | B-34675 | 20 | EP 5% | 95% | 0% |
| **Autophagy-related-13 (Atg13)** | Autophagy | RNAi | UAS-Atg13-shRNA *Short Hairpin HMS02028) | B-40861 | 20 | | | n.d. |
| **Autophagy-related-14 (Atg14)** | Autophagy | RNAi | UAS-Atg14-shRNA (Short Hairpin HMS02025) | B-40858 | 20 | PP 5%, LP 15% | 80% | 50% |
| **Autophagy-related-16 (Atg16)** | Autophagy | RNAi | UAS-Atg16-shRNA (Short Hairpin HMS01347) | B-34358 | 20 | | | n.d. |

| Gene pertubed | Biological function | Gene perturbation Type | Construct/ TRip # | Stock Number | Prepupae scored | Lethality Stage | Eclosion [%] | Flightless rate [%] |
|---|---|---|---|---|---|---|---|---|
| **Autophagy-related-17 (Atg17)** | Autophagy | RNAi | UAS-Atg17-shRNA (Short Hairpin HMS01611) | B-36918 | 20 | | | n.d. |
| **Autophagy-related-18 (Atg18)** | Autophagy | RNAi | UAS-Atg18-shRNA (Short Hairpin HMS01193) | B-34714 | 20 | | 100% | 0% |
| **Autophagy-related-2 (Atg2)** | Autophagy | RNAi | UAS-Atg2-shRNA (Short Hairpin HMS01198) | B-34719 | 0 | BP 100% | 0% | n.d. |
| **Autophagy-related-4a (Atg4a)** | Autophagy | RNAi | UAS-Atg4-shRNA (Short Hairpin HMS01482) | B-35740 | 20 | LP 2% | 98% | 78% |
| **Autophagy-related-5 (Atg5)** | Autophagy | RNAi | UAS-Atg5-shRNA (Short Hairpin HMS01244) | B-34899 | 20 | | 100% | 0% |
| **Autophagy-related-6 (Atg6)** | Autophagy | RNAi | UAS-Atg6-shRNA (Short Hairpin HMS01483) | B-35741 | 20 | LP 2% | 98% | 0% |
| **Autophagy-related-7 (Atg7)** | Autophagy | RNAi | UAS-Atg7-shRNA (Short Hairpin HMS01358) | B-34369 | 20 | | 100% | 0% |
| **Autophagy-related-8a (Atg8a)** | Autophagy | RNAi | UAS-Atg8a-shRNA (Short Hairpin HMS01328) | B-34340 | 20 | | 100% | 0% |
| **Autophagy-related-8b (Atg8b)** | Autophagy | RNAi | UAS-Atg8b-shRNA (Short Hairpin HMS01245) | B-34900 | 20 | | 100% | 0% |
| **Autophagy-related-9 (Atg9)** | Autophagy | RNAi | UAS-Atg9-shRNA (Short Hairpin HMS01246) | B-34901 | 20 | | 100% | 0% |
| **bent (bt)** | sarcomere organization | RNAi | UAS-bt-lhRNA (Long Hairpin JF01108) | B-31546 | 0 | larval | 0% | n.d. |
| **beta subunit of type I geranylgeranyl transferase (betaggt-I)** | Protein prenylation, RAS signalling | RNAi | UAS-betaggt-I-shRNA (Short Hairpin HMS01165) | B-34687 | 20 | | 100% | 0% |
| **Bruce** | apoptosis | RNAi | UAS-dBruce-RNAi | VDRC 107620 | 14 | | 100% | 0% |
| **Bruce** | apoptosis | RNAi | UAS-dBruce-RNAi | VDRC 48309 | 20 | | 95% | 0% |
| **Cadherin 99C (Cad99C)** | Cell adhesion | RNAi | UAS-Cad99C-lhRNA (Long Hairpin JF02660) | B-27510 | 20 | LP 5% | 95% | 0% |
| **Cadherin 99C (Cad99C)** | Cell adhesion | RNAi | UAS-Cad99c-shRNA (Short Hairpin HMS01451) | B-35037 | 20 | LP 10% | 90% | 0% |

| Gene pertubed | Biological function | Gene perturbation Type | Construct/ TRip # | Stock Number | Prepupae scored | Lethality Stage | Eclosion [%] | Flightless rate [%] |
|---|---|---|---|---|---|---|---|---|
| **cAMP-dependent protein kinase 1 (Pka-C1)** | protein kinase | RNAi | UAS-Pka-C1-shRNA (Short Hairpin GL00038) | B-35169 | 20 | | 100% | 0% |
| **Catalase (Cat)** | Heart morphogenesis | RNAi | UAS-Cat-shRNA (Short Hairpin HMS00990) | B-34020 | 20 | | 90% | 0% |
| **cathD** | autophagic cell death, proteolysis | RNAi | UAS-cathD-lhRNA (Long Hairpin HM05189) | B-28978 | 15 | | 100% | 0% |
| **CG13579** | G-protein couples receptor | RNAi | UAS-CG13579-lhRNA (Long Hairpin JF03059) | B-28644 | 20 | | 100% | 0% |
| **chico** | Cell size, proliferation | RNAi | UAS-chico-shRNA (Short Hairpin HMS01553) | B-36665 | 20 | | 95% | 0% |
| **chico** | Cell size, proliferation | RNAi | UAS-chico-shRNA (Short Hairpin GL00525) | B-36788 | 20 | | 95% | 0% |
| **Chro (Chromator)** | Interacts with EAST | RNAi | UAS-Chro-shRNA (Short Hairpin GL00503) | B-36084 | 30 | LP 2% | 97% | 0% |
| **coracle (cora)** | actin cytoskeleton, heart development | RNAi | UAS-cora-shRNA (Short Hairpin HMS01413) | B-35003 | 20 | | 100% | no |
| **Cysteine Proteinase 1 (Cp1)** | autophagic cell death | RNAi | UAS-Cp1-shRNA (Short Hairpin HMS02336) | B-41939 | 20 | | 100% | 0% |
| **Cysteine Proteinase 1 (Cp1)** | autophagic cell death | RNAi | UAS-Cp1-shRNA (Short Hairpin HMS00725) | B-32932 | 20 | LP 5% | 95% | 0% |
| **dawdle** | Myostatin homolog | RNAi | UAS-dawdle-shRNA (Short Hairpin HMS01110) | B-34974 | 20 | | | |
| **death executioner Bcl-2 homologue (debcl)** | apoptosis | RNAi | UAS-debcl-lhRNA (Long Hairpin JF02429) | B-27083 | 20 | | 100% | 0% |
| **Death-associated inhibitor of apoptosis 2 (Diap2)** | apoptosis | RNAi | UAS-Diap2-shRNA (Short Hairpin HMS00085) | B-34476 | 20 | | | no |
| **defective proboscis extension response 5 (dpr5)** | unknown | RNAi | UAS-dpr5-lhRNA (Long Hairpin JF03306) | B-29627 | 20 | PP 5%, LP 5% | 90% | 0% |
| **Drosophila inhibitor of apoptosis 1 (Diap1)** | negative regulator of apoptosis | Protein OE | UAS-Diap1 | B-6657 | 20 | | 100% | 0% |
| **Drosophila inhibitor of apoptosis 1 (Diap1)** | Apoptosis | RNAi | UAS-Diap1-shRNA (Short Hairpin HMS00752) | B-33957 | 20 | | | n.d. |

| Gene pertubed | Biological function | Gene perturbation Type | Construct/ TRip # | Stock Number | Prepupae scored | Lethality Stage | Eclosion [%] | Flightless rate [%] |
|---|---|---|---|---|---|---|---|---|
| **east** | Cell death, delays cell death of salivary glands & muscles | Protein OE | UAS-eastN2(1-1520)-GFP | MW | 40 | | 98% | 0% |
| **east** | Cell death, delays cell death of salivary glands & muscles | Protein OE | UAS-eastN1(1-1902)-GFP | MW | 46 | | 65% | 100% |
| **east** | Cell death, delays cell death of salivary glands & muscles | RNAi | UAS-east-shRNA Short Hairpin HMS00816) | B-33879 | 0 | BP+PP 100% | 0% | n.d. |
| **Ecdysone Receptor (EcR)** | Master regulator of metamorphosis | RNAi | UAS-EcR-RNAi | B-9326 | 1 | BP+PP 100% | 0% | n.d. |
| **Ecdysone-induced protein 63E (Eip63E)** | Metamorphosis | RNAi | UAS-Epi63E-shRNA, Short Hairpin HMS00569) | B-34075 | 20 | EP 20% | 80% | 0% |
| **Endophilin B (EndoB)** | Membrane organization | RNAi | UAS-EndoB-shRNA (Short Hairpin HMS01285) | B-34935 | 20 | | | n.d. |
| **escargot (esg)** | Transcription factor | RNAi | UAS-esg-shRNA (Short Hairpin HMS00025) | B-34063 | 20 | LP 35% | 65% | partial |
| **forkhead box, sub-group O (foxo)** | insulin receptor signalling, muscle atrophy | Protein OE | UAS-foxo | B-9575 | 0 | BP+PP 100% | 0% | n.d. |
| **forkhead box, sub-group O (foxo)** | insulin receptor signalling, muscle atrophy | RNAi | UAS-foxo-shRNA (Short Hairpin HMS00422) | B-32427 | 20 | | | n.d. |
| **GXIVsPla2** | phospholipase A2 | RNAi | UAS-GXIVsPLA2-shRNA (Short Hairpin HMS00918) | B-33961 | 34 | PP 9%, EP 26%, LP44% | 21% | 100% |
| **happyhour (hppy)** | Cell death, size and growth | RNAi | UAS-hppy-shRNA (Short Hairpin GL00185) | B-35284 | 20 | | 100% | 0% |
| **Heterogeneous nuclear ribonucleoprotein at 98DE (Hrb98DE)** | mRNA splicing | RNAi | UAS-Hrb98DE-shRNA (Short Hairpin HMS00342) | B-32351 | 20 | EP 25%, LP 40% | 35% | 100% |
| ~~**hippo (hpo)**~~ | ~~Hippo pathway, organ size, proliferation~~ | ~~RNAi~~ | ~~UAS-Hippo-RNAi~~ | ~~33614~~ | | | | ~~0%~~ |

| Gene pertubed | Biological function | Gene perturbation Type | Construct/ TRip # | Stock Number | Prepupae scored | Lethality Stage | Eclosion [%] | Flightless rate [%] |
|---|---|---|---|---|---|---|---|---|
| **hippo (hpo)** | Hippo pathway, organ size, proliferation | RNAi | UAS-hpo-shRNA (Short Hairpin GL00046) | B-35176 | 20 | | 100% | 0% |
| **immune response deficient 1 (ird1)** | Autophagy | RNAi | UAS-ird1-shRNA (Short Hairpin GL00085) | B-35209 | 20 | | | 0% |
| **Insulin-like receptor (InR)** | cell survival, body and organ size | RNAi | UAS-InR-shRNA (Short Hairpin GL00139) | B-35251 | 20 | PP 5%, EP 80%, LP 10% | 5% | yes |
| **Lola-like (lolal)** | chromatin silencing | RNAi | UAS-lolal-shRNA (Short Hairpin GLV21087) | B-35722 | 20 | LP 5% | 95% | 0% |
| **Lst8** | Cell size, TOR signalling | RNAi | UAS-Lst8-shRNA (Short Hairpin HMS01350) | B-34361 | 20 | | | |
| **Megator (Mtor)** | mitosis, chromatin, nuclear pore complex | RNAi | UAS-Mtor-shRNA (Short Hairpin HMS00735) | B-32941 | 0 | BP+PP 100% | 0% | n.d. |
| **misfire (mfr)** | Ferlin domain | RNAi | UAS-mfr-shRNA (Short Hairpin GLV21054) | B-35689 | 20 | LP 25% | 75% | No |
| **modifier of rpr and grim, ubiquitously expressed (morgue)** | cell death | RNAi | UAS-morgue-RNAi | VDRC 11090 | 15 | LP 7% | 93% | 0% |
| **Molecule interacting with CasL (Mical)** | sarcomere organization, actin depolymerization | RNAi | UAS-Mical-lhRNA (Long Hairpin JF01625) | B-31148 | 20 | | 100% | No |
| **Msp300 (Muscle specific protein 300)** | muscle differentiation | RNAi | UAS-Msp300-shRNA (Short Hairpin HMS00368) | B-32377 | 20 | | | n.d. |
| **Muscle protein 20 (Mp20)** | muscle differentiation | RNAi | UAS-Mp20-shRNA (Short Hairpin HMS00630) | B-34963 | 20 | | | n.d. |
| **muscleblind (mbl)** | muscle development | RNAi | UAS-mbl-lhRNA (Long Hairpin JF03264) | B-29585 | 20 | | | n.d. |
| **Myo31DF (Myosin 31DF)** | movement of organelles | RNAi | UAS-Myo31DF-shRNA (Short Hairpin HMS00928) | B-33971 | 20 | | 100% | 0% |
| **myotubularin (mtm)** | myotubularin | RNAi | UAS-mtm-shRNA (Short Hairpin HMS01806) | B-38339 | 20 | | | n.d. |
| **p53** | cell death | RNAi | UAS-p53-shRNA (Short Hairpin GL01032) | B-36814 | 21 | | 100% | 0% |
| **p53** | cell death | RNAi | UAS-p53-shRNA (Short Hairpin HMS02286) | B-41720 | 20 | EP 5% | 95% | 0% |

| Gene pertubed | Biological function | Gene perturbation Type | Construct/ TRip # | Stock Number | Prepupae scored | Lethality Stage | Eclosion [%] | Flightless rate [%] |
|---|---|---|---|---|---|---|---|---|
| **pelle (pll)** | cell death | RNAi | UAS-pll-shRNA (Short Hairpin HMS01213) | B-34733 | 20 | | 100% | 0% |
| **pelle (pll)** | cell death | RNAi | UAS-pll-shRNA (Short Hairpin GL00150) | B-35577 | 20 | | 100% | 0% |
| **Phosphofructokinase (Pfk)** | Metabolism | RNAi | UAS-Pfk-shRNA (Short Hairpin GL00298) | B-36782 | 20 | | 100% | 0% |
| **Phosphofructokinase (Ppfk)** | Metabolism | RNAi | UAS-Pfk-shRNA (Short Hairpin HMS01324) | B-34336 | 4 | few prepupae | 100% | 100% |
| **Phosphotidylinositol 3 kinase 59F (Pi3K59F)** | autophagy | RNAi | UAS-Pi3K59F-shRNA (Short Hairpin GL00175) | B-36056 | 20 | | | n.d. |
| **Polycomblike (Pcl)** | Transcription factor | RNAi | UAS-Pcl-shRNA (Short Hairpin HMS00897) | B-33946 | 20 | | 100% | 0% |
| **Rac1** | Muscle cell differentiation | Protein OE (CA) | UAS-Rac1.V12 | B-6291 | 0 | BP 100% | 0% | n.d. |
| **Rac1** | Muscle cell differentiation | Protein OE (DN) | UAS-Rac1.N17 | B-6292 | 10 | EP 50%, LP 17% | 33% | 100% |
| **rapamycin-insensitive companion of Tor (rictor)** | TOR pathway | RNAi | UAS-rictor-shRNA (Short Hairpin GL00544) | B-36584 | 20 | LP 5% | 95% | 33% |
| **rapamycin-insensitive companion of Tor (rictor)** | TOR pathway | RNAi | UAS-rictor-shRNA (Short Hairpin HMS01588) | B-36699 | 20 | | 100% | 0% |
| **Ras** | cell death and proliferation | Protein OE (DN) | UAS-Ras.N17 | B-4846 | 20 | | 100% | 0% |
| **Ras homolog enriched in brain ortholog (Rheb)** | TOR pathway | Protein OE | UAS-Rheb (3rd) | B-9689 | 15 | | 100% | 100% |
| **Ras homolog enriched in brain ortholog (Rheb)** | TOR pathway | RNAi | UAS-Rheb-shRNA (Short Hairpin HMS00923) | B-33966 | 32 | LP 19% | 81% | 100% |
| **Ras oncogene at 64B (Ras64B)** | cell death and proliferation | Protein OE (CA) | UAS-Ras64B.V14 | B-2025 | 9 | | 100% | 100% |
| **Ras oncogene at 85D (Ras85D)** | cell death and proliferation | Protein OE | UAS-Ras85D | B-5788 | 20 | | 100% | 0% |

| Gene pertubed | Biological function | Gene perturbation Type | Construct/ TRip # | Stock Number | Prepupae scored | Lethality Stage | Eclosion [%] | Flightless rate [%] |
|---|---|---|---|---|---|---|---|---|
| **Ras85D** | cell death and proliferation | Protein OE (CA) | UAS-Ras.V12 | B-4847 | 0 | BP 100% | 0% | n.d. |
| **Ras85D** | cell death and proliferation | Protein OE (DN) | UAS-Ras85D.N17 | B-4845 | 20 | | 100% | 0% |
| **reaper (rpr)** | cell death | Protein OE | UAS-rpr | B-5824 | 0 | BP 100% | 0% | n.d. |
| **Rho1** | actin cytoskeleton | Protein OE (DN) | UAS-Rho1.N19 | B-7328 | 10 | LP 70% | 30% | 0% |
| **Rm62** | RNA interference, RNA helicase | RNAi | UAS-Rm62-shRNA (Short Hairpin HMS00144) | B-34829 | 54 | PP 6%, EP 2%, LP 54% | 39% | 100% |
| **RPS6-p70-protein kinase (S6k)** | protein translation | RNAi | UAS-S6k-shRNA (Short Hairpin GL01327) | B-41895 | 20 | | | n.d. |
| **salvador** | Hippo pathway, organ size, proliferation | RNAi | UAS-salvador-shRNA (Short Hairpin HMS00760) | B-32965 | 20 | | | 0% |
| **scarface (scaf)** | proteolysis | RNAi | UAS-scaf-lhRNA (Long Hairpin JF03318) | B-29386 | 20 | | 100% | 0% |
| **seizure (sei)** | potassium channel | RNAi | UAS-sei-lhRNA (Long Hairpin JF01474) | B-31681 | 20 | | 100% | 0% |
| **Skeletor** | spindle matrix | RNAi | UAS-Skeletor-lhRNA (Long Hairpin JF01407) | B-31622 | 20 | | 100% | 0% |
| **small ribonucleoprotein particle U1 subunit C (snRNP-U1-C)** | mRNA splicing | RNAi | UAS-snRNP-U1-C-shRNA (Short Hairpin HMS00137) | B-34822 | 0 | BP 100% | 0% | n.d. |
| **spalt-major (salm)** | Muscle cell differentiation, myofibre switch | RNAi | UAS-salm-RNAi (Short Hairpin HMS00594) | B-33714 | 20 | | 100% | 0% |
| **Target of rapamycin (TOR)** | TOR pathway, cell size control | Protein OE | UAS-TOR | B-7012 | 20 | PP 85%, EP 10%, LP 5% | 0% | n.d. |
| **Target of rapamycin (TOR)** | TOR pathway, cell size control | Protein OE (DN) | UAS-TOR-TED | B-7013 | 51 | 22% EP, 27% LP | 51% | 20% |
| **Target of rapamycin (TOR)** | TOR pathway, cell size control | RNAi | UAS-TOR-shRNA (Short Hairpin GL00156) | B-35578 | 20 | LP 20% | 80% | partial (29%) |

| Gene pertubed | Biological function | Gene perturbation Type | Construct/ TRip # | Stock Number | Prepupae scored | Lethality Stage | Eclosion [%] | Flightless rate [%] |
|---|---|---|---|---|---|---|---|---|
| **Target of rapamycin (TOR)** | TOR pathway, cell size control | RNAi | UAS-TOR-shRNA (Short Hairpin HMS01114) | B-34369 | 20 | | 100% | 0% |
| **Target of rapamycin (TOR)** | TOR pathway, cell size control | RNAi | UAS-TOR-shRNA (Short Hairpin HMS00904) | B-33951 | 7 | PP 100% | 0% | n.d. |
| **target of rapamycin (TOR)-associated protein (raptor)** | TOR signalling | RNAi | UAS-raptor-shRNA (Short Hairpin HMS00124) | B-34814 | 15 | PP 100% | 0% | n.d. |
| **terribly reduced optic lobes (trol)** | | RNAi | UAS-trol-shRNA (Short Hairpin GL01153) | B-42783 | 20 | | | n.d. |
| **thin, another B-box affiliate (tn)** | muscle development | RNAi | UAS-tn-shRNA (Short Hairpin HMS02508) | B-42826 | 20 | | | 0% |
| **Tsc1** | TOR pathway, cell size control | RNAi | UAS-Tsc1-shRNA (Short Hairpin GL00012) | B-35144 | 20 | | 80% | 22% |
| **Tsc2, gigas (gig)** | TOR pathway, cell size control | RNAi | UAS-Tsc2-shRNA (Short Hairpin HMS01217) | B-34737 | 20 | PP 20%, EP 5%, LP 15% | 60% | 64% |
| **Tsc2, gigas (gig)** | TOR pathway, cell size control | RNAi | UAS-Tsc2-shRNA (Short Hairpin GL00321) | B-35401 | 21 | | 100% | 6% |
| **Ubiquitin conjugating enzyme (UbcD6)** | Proteolysis | RNAi | UAS-UbcD6-shRNA (Short Hairpin GL00405) | B-35476 | 20 | LP 5% | 95% | 0% |
| **Unc-89** | sarcomere organization, muscle differentiation | RNAi | UAS-Unc-89-shRNA (Short Hairpin HMS00963) | B-34000 | 20 | | 100% | 0% |
| **UV-resistance associated gene (UVRAG)** | Autophagy, Cell growth | RNAi | UAS-Uvrag-shRNA (Short Hairpin HMS01357) | B-34368 | 20 | | | n.d. |
| **warts (wts)** | Hippo pathway, organ size, proliferation | RNAi | UAS-wts-shRNA (Short Hairpin HMS00026) | B-34064 | 20 | | 100% | 0% |
| **yorkie (yki)** | Hippo pathway, organ size, proliferation | RNAi | UAS-yki-shRNA (Short Hairpin HMS00041) | B-34067 | 15 | LP 7% | 93% | 100% |
