# Supplementary figures and images for: Live imaging of muscle histolysis in Drosophila metamorphosis
Source: BMC Dev Biol. 2016 May 4;16:12. doi: 10.1186/s12861-016-0113-1 (PMC4855724; doi:10.1186/s12861-016-0113-1)

(A) Prepupa

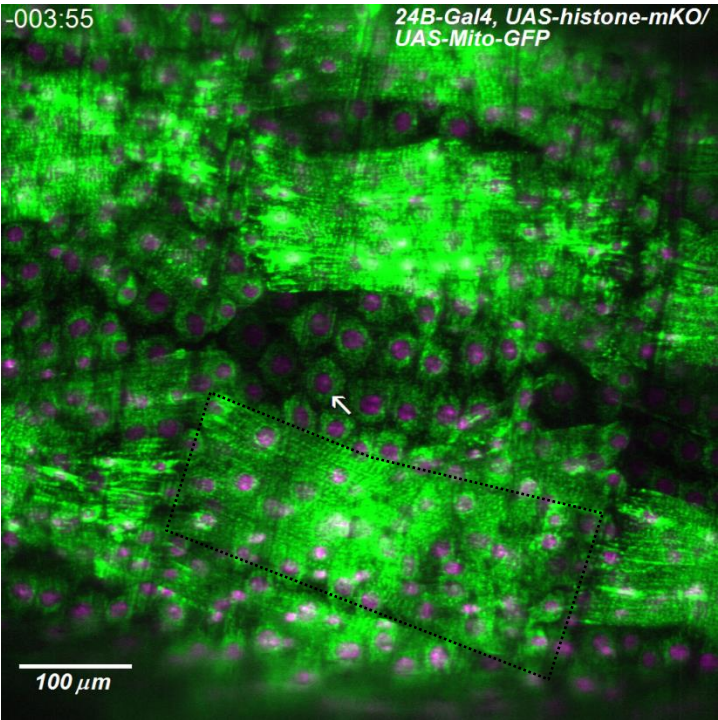

(B) Pupa

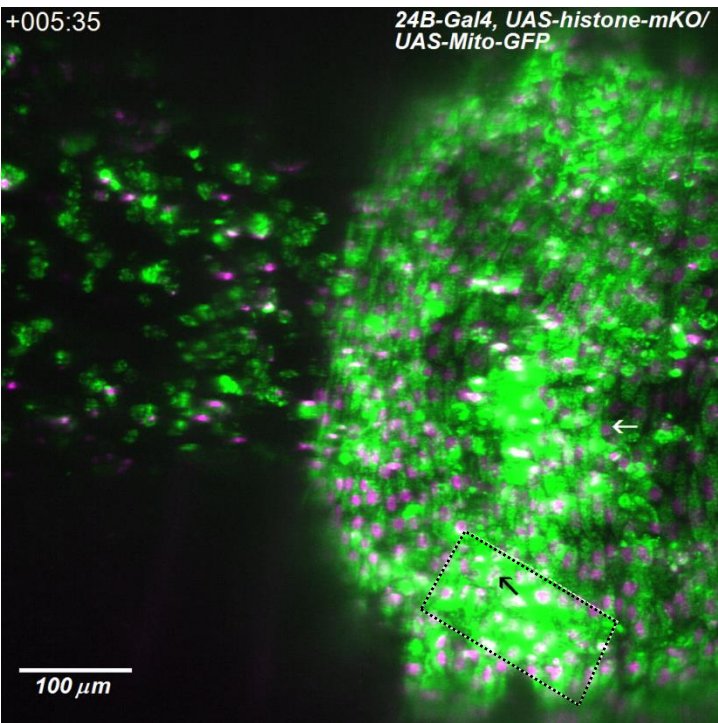

Additional File 2: Figure S1. Expression of the 24B-GAL4 driver in metamorphosis.

Supplement: Additional file 2: Figure S1. — Expression of the 24B-GAL4 driver in metamorphosis. In (A) prepupae and (B) pupae, the mesodermal 24B-Gal4 driver is expressed in multi-nucleated muscles (outlined in black) and more apically located mono-nucleated cells (white arrow). Cells were labelled with the mitochondrial marker UAS-Mito-GFP (green) and UAS-histone-mKO (magenta). (B) During pupation, mono-nucleated cells (white arrow) undergo PCD and show nuclear fragmentation, while nuclei (black arrow) in muscles undergoing histolysis, like the DIOM2s (outlined in black), condense without showing fragmentation (see Fig. 2). (PDF 218 kb) [file 12861_2016_113_MOESM2_ESM.pdf]
