# Supplementary material for: Live imaging of muscle histolysis in Drosophila metamorphosis
Source: BMC Dev Biol. 2016 May 4;16:12. doi: 10.1186/s12861-016-0113-1 (PMC4855724; doi:10.1186/s12861-016-0113-1)
Supplement: Additional file 3: Figure S2. — Two different Cp1-shRNAs silence expression of a Cp1-mKO2 reporter during prepupal (A) and pupal (B) stages. The construct HMS00725 (second row) was used in most experiments. (PDF 6018 kb) [file 12861_2016_113_MOESM3_ESM.pdf]

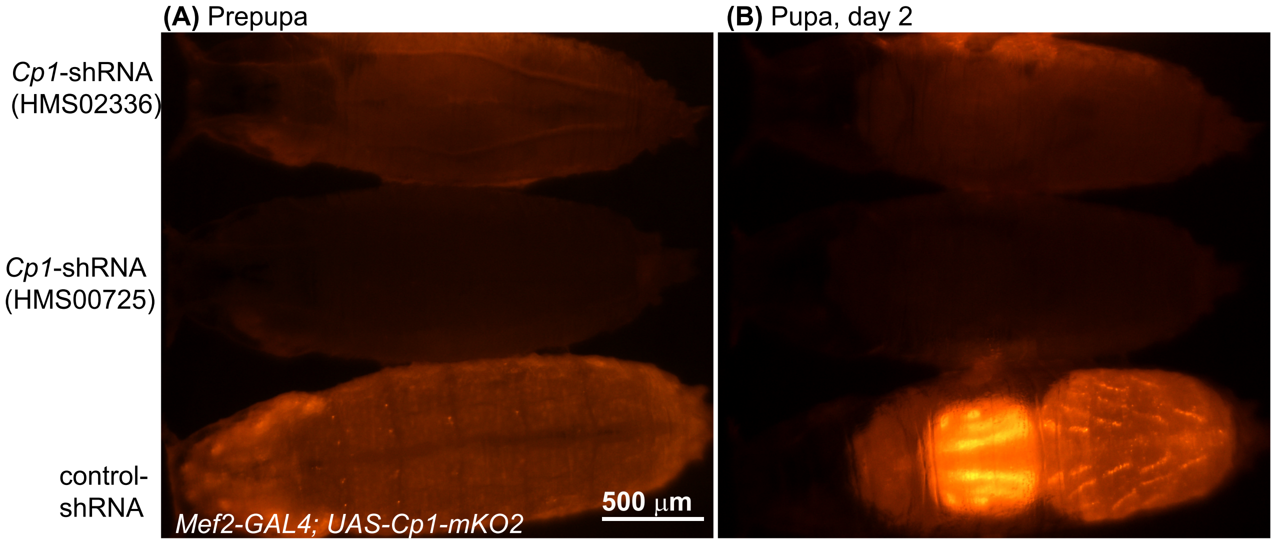

Additional file 3: Figure S2. Two different *Cp1*-shRNAs repress expression of a Cp1-mKO2 reporter during prepupal and pupal stages.
